# An ESKD Patient with a Severe, Progressive Headache

**DOI:** 10.34067/KID.0000000714

**Published:** 2025-06-26

**Authors:** Isobel Dunbabin, Eoin Daniel O’Sullivan

**Affiliations:** 1 Kidney Health Service, Royal Brisbane and Women's Hospital, Herston, Queensland, Australia; 2 Institute for Molecular Bioscience, The University of Queensland, Herston, Queensland, Australia; 3 QIMR Berghofer Medical Research Institute, Brisbane, Queensland, Australia

**Keywords:** dialysis, hyperparathyroidism, mineral metabolism, renal osteodystrophy

## Abstract

**Figure:**
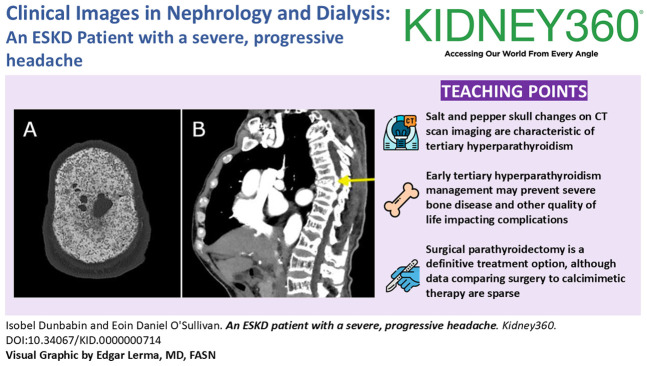

A 55-year-old man with dialysis-dependent ESKD and tertiary hyperparathyroidism presented with severe headache, which had been progressively worsening over weeks. He had been receiving hemodialysis treatments for 20 years. He had developed tertiary hyperparathyroidism 13 years before presentation, had chosen not to undergo parathyroidectomy, and opted for therapy with maximally tolerated cinacalcet. On physical examination, he was tender to palpation across his facial bones, zygomatic arch, and temporal bones. Thoracic kyphosis was noted, but the patient did not have any spinal tenderness. The rest of his examination was unremarkable.

A computed tomography (CT) scan of the head demonstrated a mottled appearance characteristic of salt and pepper skull (Figure 1A), and a CT scan of the spine demonstrated changes consistent with rugger jersey spine (Figure 1B). His serum parathyroid hormone (PTH) level was markedly elevated at 508 pmol/L (4792 pg/ml) with an elevated serum corrected calcium of 2.73 mmol/L (10.95 mg/dl), indicative of poorly controlled tertiary hyperparathyroidism. On review, it was noted that his serum PTH levels had been grossly elevated (>200 pmol/L) for 11 years before presentation. He was referred for an elective parathyroidectomy for definitive management.

**Figure 1:**
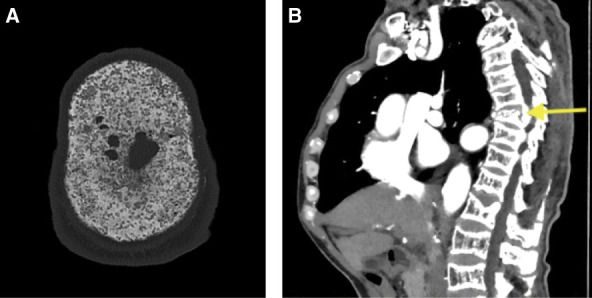
**CT demonstrates changes consistent with severe tertiary hyperparathyroidism.** (A) CT scan imaging demonstrates a mottled appearance of skull calvaria consistent with salt and pepper skull. (B) CT scan demonstrates rugger jersey spine and compression fracture. CT, computed tomography.

Salt and pepper skull is a distinctive radiological description of advanced renal osteodystrophy. This can be seen in advanced hyperparathyroidism, where excessive bone resorption results in a mottled appearance of the calvaria on CT scan imaging. The target range for serum PTH in ESKD is uncertain, but typically two- to nine-fold the upper limit of normal is advised. Cinacalcet is an oral calcimimetic agent with a role in lowering elevated serum PTH levels. When compared against placebo, cinacalcet potentially lowered fracture rates but did not modify cardiovascular risk in the initial Evaluation of Cinacalcet Hydrochloride Therapy to Lower Cardiovascular Events trial analysis.^1,2^ Secondary analysis has suggested there may be an older subgroup of patients who have some cardiovascular benefit.^3^

It is not known whether cinacalcet or surgical parathyroidectomy has differential effects on bone parameters and related outcomes in this setting. In terms of clinical trial data, a recent phase two trial in patients with ESKD on peritoneal dialysis suggests higher bone mineral density in those who undergo parathyroidectomy.^4^ A pilot randomized trial showed equivalence across multiple surrogate cardiovascular disease measurements, biochemical end points, and quality-of-life measures.^5^

## Teaching Points

- Salt and pepper skull changes on CT scan imaging are characteristic of tertiary hyperparathyroidism.
- Early tertiary hyperparathyroidism management may prevent severe bone disease and other quality-of-life–affecting complications.
- Surgical parathyroidectomy is a definitive treatment option, although data comparing surgery with calcimimetic therapy are sparse.
